# Influence of a six month endurance exercise program on the immune function of prostate cancer patients undergoing Antiandrogen- or Chemotherapy: design and rationale of the ProImmun study

**DOI:** 10.1186/1471-2407-13-272

**Published:** 2013-06-03

**Authors:** Philipp Zimmer, Elke Jäger, Wilhelm Bloch, Eva Maria Zopf, Freerk T Baumann

**Affiliations:** 1 Department of Molecular and Cellular Sport Medicine, Institute of Cardiovascular Research and Sport Medicine, German Sport University Cologne, Am Sportpark Müngersdorf 6, Köln 50933, Germany; 2 Department for Oncology and Hematology, Clinic Northwest, Steinbacher Hohl 2-26, Frankfurt am Main 60488, Germany

**Keywords:** Exercise, Prostate cancer, Immune function

## Abstract

**Background:**

Exercise seems to minimize prostate cancer specific mortality risk and treatment related side effects like fatigue and incontinence. However the influence of physical activity on the immunological level remains uncertain. Even prostate cancer patients undergoing palliative treatment often have a relatively long life span compared to other cancer entities. To optimize exercise programs and their outcomes it is essential to investigate the underlying mechanisms. Further, it is important to discriminate between different exercise protocols and therapy regimes.

**Methods/Design:**

The ProImmun study is a prospective multicenter patient preference randomized controlled trial investigating the influence of a 24 week endurance exercise program in 80–100 prostate cancer patients by comparing patients undergoing Antiandrogen therapy combined with exercise (AE), Antiandrogen therapy without exercise (A), Chemotherapy with exercise(CE) or Chemotherapy without exercise (C). The primary outcome of the study is a change in prostate cancer relevant cytokines and hormones (IL-6, MIF, IGF-1, Testosterone). Secondary endpoints are immune cell ratios, oxidative stress and antioxidative capacity levels, VO_2 peak_, fatigue and quality of life. Patients of the intervention group exercise five times per week, while two sessions are supervised. During the supervised sessions patients (AE and CE) exercise for 33 minutes on a bicycle ergometer at 70-75% of their VO_2 peak_. To assess long term effects and sustainability of the intervention two follow-up assessments are arranged 12 and 18 month after the intervention.

**Discussion:**

The ProImmun study is the first trial which primarily investigates immunological effects of a six month endurance exercise program in prostate cancer patients during palliative care. Separating patients treated with Antiandrogen therapy from those who are additionally treated with Chemotherapy might allow a more specific view on the influence of endurance training interventions and the impact of different therapy protocols on the immune function.

**Trial registration:**

German Clinical Trials Register: DRKS00004739

## Background

Within the last decade, an increasing number of studies were able to demonstrate that physical activities in general, as well as defined and controlled exercise programs are beneficial for prostate cancer patients. Kenfield and colleagues [1] provided evidence that regular physical activity may reduce overall mortality and prostate cancer specific mortality. Other well designed studies show a positive influence of exercise interventions regarding treatment related side effects like urinary incontinence after surgery [2,3] and body composition changes during Antiandrogen therapy [4]. Positive effects of exercise on general cancer- and cancer therapy associated symptoms like fatigue could have been detected as well [5]. Finally physical activity increases the endurance capacity, muscular strength and quality of life in prostate cancer patients independent of their stage of disease [6-9].

Most of the underlying mechanisms leading to the described desirable effects of physical activity are poorly investigated. In order to optimize the outcome of exercise interventions, future research has to reveal the systemic influence of physical activity on the molecular and cellular level. As in drug development, dose-effect relationships will play a key role in creating exercise programs. Since exercise programs and their systemic effects differ enormously (e.g. intensities, frequencies and type of exercise), it is essential to investigate them separately. This should also be considered when regarding exercise interventions with different cancer entities and during different medical treatment regimes. A first approach to learn more about the cellular effects of a defined resistance exercise program in prostate cancer patients undergoing Antiandrogen therapy has been presented by Thorsen et al. [10]. However this study focuses on the influence of strength training on muscle tissue and body composition.

Knowledge about the impact of physical activity on the tumor, tumor relevant growth factors and the immune system is still rudimental. In a pilot study we investigated the influence of a 1408 km bicycle tour on Testosterone, Interleukin 6 and PSA levels in prostate cancer patients. In accordance with the results by Segal et al. [6], we found a decrease in Testosterone levels whereas the other Parameters did not change [11]. Only a few studies with humans have focused on the impact of exercise interventions on Cytokine levels or immune cells in cancer patients. Furthermore, these studies primarily focused on breast cancer patients [12-14]. In order to present possible connections between prostate cancer, exercise and the genesis or the progression of the disease, we would like to highlight three animal studies.

Teixeira et al. [15] were able to show that endurance exercise has the potential to induce changes in sex hormone levels and sex hormone receptors in the ventral prostate of healthy rats. Exercising animals showed increased levels of Corticotestosterone, Dihydrotestosterone, Testosterone and Estrogen receptors whereas Androgen receptors decreased. In contrast to the control group, exercising animals showed a modified proliferation-apoptosis ratio with a shift toward apoptosis. Jones and colleagues [16] presented a prostate cancer mouse model. In this study, tumor growth rates did not differ between animals in the exercising group and those in the control group. Interestingly a significant reduction of the expression of prometastasic genes could be observed in the exercising animals. Exercise also seemed to stabilize the tumor vascular system, leading to an improved endothelial barrier which may constrict the migration of metastatic cells. Finally Zheng et al. [17] suggest that exercise may inhibit the progression of advanced prostate cancer cells, leading to a delayed Androgen independency of the disease. Exercising mice showed a 38% decrease in mitotic cell/caspase 3 positive tumor cell ratio and featured a reduced increase of IL-6. IL-6 is discussed to be needed by the tumor cells in order to become Androgen independent. Regarding the described effects, exercise was even more effective when it was combined with a caffeine substitution. Chronic and acute exercise induced IL-6 alterations are also well described in humans [18].

Within the ProImmun trial we would like to focus on the influence of a six month supervised endurance exercise program on prostate cancer relevant cytokines and immune function in patients with advanced prostate cancer undergoing Antiandrogen therapy or Antiandrogen therapy in combination with chemotherapeutic agents. As described above [12-14] endogenous tumor defense might be stimulated by physical activity, and therefore lead to an improved outcome in terms of relapse- and mortality risk. We expect that an enhanced immune function may help to stabilize even advanced stages of prostate cancer.

## Methods/Design

**Figure 1:**
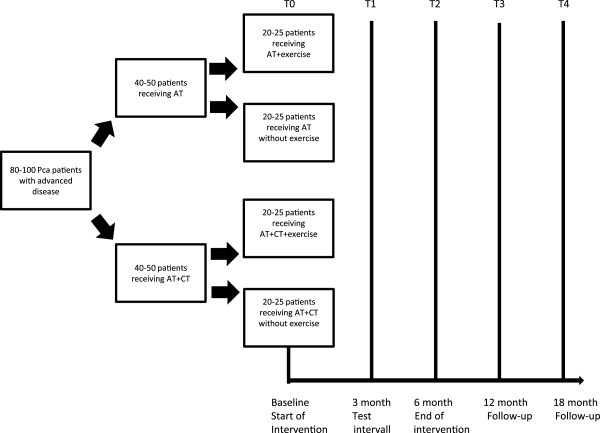
Study design with patient enrolment and measurement time points.

The ProImmun study was planned as a four arm, prospective multicenter preference randomized trial. For this purpose a collaboration consisting of the Department of Molecular and Cellular Sports Medicine of the German Sport University Cologne, the community of private practicing urologists at cologne (KCU) and the Department of Oncology and Hematology of the Hospital Northwest at Frankfurt was installed. All patients will provide written informed consent prior to participation. The study protocol has been approved by the ethics committee of the German Sport University Cologne and the ethics committee of the Krankenhaus Nordwest at Frankfurt (Figure 1).

### Subjects and sample size calculation

Regarding changes in cytokine levels and immune cell compartments through physical activity no effect size is known for patients suffering from advanced prostate cancer. Even for other cancer entities and diseases in combination with exercise programs effect sizes of the named factors are not alienable due to different therapies or varying exercise programs. This circumstance would lead to an imprecise, ambiguous sample size calculation. Therefor we decided to choose a sample size which enables us to perform descriptive statistic tests (e.g. T-tests, variance analyses) with 20–25 patients in each of the four groups.

### Inclusion and exclusion criteria (Table 1)

**Table 1:** Inclusion and exclusion criteria

**Inclusion criteria**	**Exclusion criteria**
• Patients with advanced prostate cancer receiving Antiandrogen therapy for at least 4 weeks or	• Anemia > 8 g/dl
	• Platelet count ≤ 10000/μl
	• COPD
• Patients with advanced prostate cancer receiving Antiandrogen therapy in combination with chemotherapy (baseline test before chemotherapy)	• CNS Metastasis
	• Epilepsy
	• Planned Surgery
	• Heart failure (NYHA III-IV)
• Hemoglobin > 9 g/dl	• Coronary disease
• Ejection fraction ≥ 60%	• Therapy refractory Hypertension
• Forced expiratory volume in one second ≥ 50%	• Other internistic diseases that rule out exercise
	• Orthopedic handicaps that rule out exercise
• Age ≥ 18 years	• Psychological problems which are critical in view of the responsible medical doctor
• Expectation of life ≥ 6 month	
• Written consent	
• Inconspicuous ultrasound of the patients heart	
• Inconspicuous ECG (rest and stress)	

### Recruitment

In a first step, private urologists in cologne and physicians at the Department of Oncology and Hematology of the Hospital Northwest in Frankfurt screen all potential study participants and provide oral information about the intervention study. If a patient is interested, assigned consent is passed on the local study coordination, which will contact the patient. In a second step, agreeing subjects receive written information material and are invited for a preliminary conversation and baseline testing. Prior to baseline testing, patients have to provide written consent to participate in the study.

### Randomization

In accordance with the principles of a patient preference trial, only patients who do not have a strong group preference will be randomized into either the exercise or the control group using the RITA randomization software (STATSOL.de) [19-21].

### Intervention

Subjects, participating in the intervention group exercise five times per week for six months. Two exercise sessions per week are supervised. During these sessions, patients exercise for 33 minutes on a bicycle ergometer (ergoline, Bitz). After a three minute warm up, individuals exercise for 25 minutes at 70-75% of their VO_2 peak_, followed by a 5 minute cool-down. The other three exercise sessions per week are conducted home based. Patients are asked to exercise at for least 15 minutes per session and complete an exercise diary. Participants can choose their preferred type of endurance exercise (e.g. walking, nordic-walking, swimming). In order to control the home based sessions, patients are informed about their exercising heart rate range according to their preferred type of endurance exercise. The exercise intensities comply with those of the supervised sessions.

### Measurement time points

All outcomes of the study will be assessed five times in each group. After the initial baseline testing (T0), participants are tested after three month (T1). This examination is also used to readjust the intensity of the exercise program in the intervention group. The third assessment is arranged after the six month intervention (T3). Two follow-up measurements are conducted six month (T4) and twelve month (T5) after the end of the intervention.

### Primary endpoint

*Cytokine/hormone levels* IL-6, Macrophage migration inhibiting factor (MIF), Testosterone and Insluin like growth factor 1 (IGF-1) levels will be measured via ELISA as soon as the study is completed. Therefore, venous blood samples will be collected and frozen away. All named mediators seem to be involved in tumor growth and disease progression. Additionally these cytokines and hormones can be influenced by physical activity [22-25].

### Secondary enpoints

#### Immune function

To evaluate the immune function, flow cytometer analyses using a 4 four color BD FACS Array Cytometer are carried out at all time points. Different cluster of differentiation (CD) antibodies, including CD3, CD4, CD8, CD16, CD19, CD25, CD45, CD127 are used to evaluate the number of diverse T-cell compartments, B- and NK-cells.

#### Oxidative stress/Antioxidative capacity

On the one hand, raised oxidative stress levels have been described to be involved in the development of chronic diseases, e.g. cancer, and disease progression [26]. On the other hand, the effects of some chemotherapeutic drugs are based on the induction of oxidative stress [27]. Physical activity seems to influence oxidative stress levels and antioxidative capacity in prostate cancer patients [28]. Within the ProImmun trial general oxidative stress levels and antioxidative capacity are measured in peripheral blood samples using a colorimetric procedure (FORMplus, Incomat, Glashütten, Germany).

#### Endurance capacity

In order to measure endurance capacity, a modified WHO bicycle spiroergometer test is performed at each measuring time point named above. Patients start cycling at 30 Watt while the power increases by 15 Watt every two minutes. Patients work out until respiratory exhaustion sets in. The VO_2 peak_ (highest O_2_ consumption during the test) is used to control the exercise intervention and to assess the endurance capacity.

#### Physical activity levels

Physical activity levels are assessed by two different methods. As in most exercise interventions we decide to apply an evaluated German questionnaire (Freiburger Questionaire of Physical Activity) which identifies MET-scores [29]. Additionally all patients receive an ADL-monitor (Sensewear, Bodymedia), combining pedometer data, changes in temperature flow and galvanic skin response. Patients wear the ADL-monitors four times during the study period, always for one week.

#### Psychological and psycho-social assessments

Aside from investigating of physiological factors, we are interested in the influence of the intervention on Quality of life, prostate cancer specific problems, like incontinence and fatigue. Therefor the EORTC-QLQ-C30, its prostate cancer specific module PR-25 and the Multidemensional Fatigue Inventory (MFI-20) are full filled by the patients to all named time points [30-32].

## Discussion

The ProImmun trial is probably the first attempt to investigate the influence of a well-defined and practicable exercise intervention on the immune system of patients with advanced prostate cancer. It may provide several new hints regarding the impact of an endurance exercise program on cytokine levels, immune function and oxidative stress levels. Combining these data with those of disease progression and cancer (therapy) related symptoms like fatigue, may help to obtain new mechanistic insights. Even if these hints will not provide detailed information about downstream mechanism (e.g. signal transduction), they could serve as an “ door opener” for further research.

Despite the exercise program, our study design has the potential to indicate whether different therapy protocols have a different impact on the reported immunological parameters. To the best of our knowledge, so far only one study has focused on the influence of exercise on a wide range of cytokine levels in cancer patients [22]. However individuals who participated in this comparably short interventional study, exercised with relatively moderate intensities. Furthermore, the participants were breast cancer patients who exercised after having completed medical therapy. Finally, changes in cytokine levels were only combined with psycho-social parameters (e.g. Fatigue questionnaires) [33].

Separating patients who are treated with Antiandrogen therapy from those who are additionally treated with Chemotherapy may help to understand, whether the intervention has the same effect in both groups. Therewith, we get us one step closer to the final aim of cancer (stage) specific exercise programs. In order to gain more knowledge about the mode of action and dose-effect relationships, further studies need to consider different kind of exercise intensities and exercise types as presented by Santa Mina and colleagues [34].

Finally the two follow-up measurements will provide information regarding the sustainability of the exercise program. Since even patients with advanced prostate cancer have a relatively long life expectancy, we will investigate whether the intervention leads to long-term changes regarding all endpoints.

In a next step, scientists should start combining these factors. From a more experimental point of view, future research also has to focus on the influence of different exercise types and intensities on the tumor itself. It will be challenging to transfer the results of these studies on human beings and generate applicable exercise programs, as mentioned in the introduction section [15-17]. In contrast to other studies [35,36] the intensity and frequency of our endurance exercise intervention (75% of VO_2 peak_) is relatively high. Comparing the outcomes of our study to those of other endurance exercise interventions, e.g. walking programs, may allow at least first conclusions regarding the impact on outcomes like fatigue and quality of life.

For most patients, quality of life and other psycho-social factors are important motivational aspects to participate in exercise programs. Presumably the positive influence on these factors can only partly or indirectly be explained by physiological parameters. However, since the knowledge about physical activity, prostate cancer disease progression and prostate cancer specific mortality risk is increasing [1], it will be a major concern to optimize and specify exercise programs. Therefore it is essential to learn more about exercise induced effects on tumor competitive immune cells and tumor-host relevant mediators like cytokines.

## Abbreviations

NYHA: New York Heart Association; MIF: Macrophage migration inhibiting factor; ECG: Electrocardiogram; IL-6: Interleukin 6; IGF-1: Insulin like Growth Factor; CD: Cluster of Differentiation; ELISA: Enzyme-Linked Immunosorbent Assay; FORMplus: Free Oxygen Radicals Monitors; EORTC-QLQ-C30: European Organization for the Research and Treatment of Cancer- Quality of Life Questionnaire-Cancer; EORTC-QLQ-PR25: European organization for the research and treatment of cancer- quality of life questionnaire- prostate cancer; MFI-20: Multidimensional Fatigue Inventory; WHO: World Health Organization; VO2 peak: Highest O_2_ consumption during the test; MET: Metabolic equivalent.

## Competing interests

The authors declare that they have no competing interests.

## Authors’ contributions

FTB and EJ initiated the project. FTB, EJ and WB direct the study. FTB, EJ and PZ wrote the study protocol. PZ and EMZ provide access to the patients and perform laboratory analyzes. FTB and PZ will implement the protocol and will perform statistical analyzes. All authors read and approved the final script.

## Pre-publication history

The pre-publication history for this paper can be accessed here:

http://www.biomedcentral.com/1471-2407/13/272/prepub
